# Lymphocyte-to-Monocyte Ratio Is the Independent Prognostic Marker of Progression in Patients Undergoing BCG-Immunotherapy for Bladder Cancer

**DOI:** 10.3389/fonc.2021.655000

**Published:** 2021-03-26

**Authors:** Mateusz Adamkiewicz, Piotr Bryniarski, Maksymilian Kowalik, Bartłomiej Burzyński, Paweł Rajwa, Andrzej Paradysz

**Affiliations:** ^1^ Department of Urology, Medical University of Silesia, Zabrze, Poland; ^2^ Department of Rehabilitation, Faculty of Health Sciences, Medical University of Silesia, Katowice, Poland

**Keywords:** lymphocyte-to-monocyte ratio, neutrophil-to-lymphocyte ratio, platelet-to-lymphocyte ratio, bladder cancer, transurethral resection of bladder tumor, Bacillus Calmet-Guiren immunotherapy

## Abstract

**Introduction:**

Transurethral resection of bladder tumor with subsequent BCG immunotherapy is the current gold standard in the treatment of high risk and some medium-risk non-muscle invasive bladder cancer. Clinical factors like stage, grade, age and gender are well-know predictors of progression to muscle-invasive bladder cancer. In recent years novel hematological biomarkers were shown to be independent predictors of progression. This study aimed to evaluate which of these novel markers has the highest prognostic value of progression in patients with bladder cancer receiving BCG immunotherapy.

**Materials and methods:**

We retrospectively analyzed the data of 125 patients with non-muscle invasive bladder cancer who received BCG immunotherapy. Of these, 61 progressed to muscle-invasive disease or had high-grade recurrence. These patients were compared with the group who did not progress (n = 64). Clinical data including stage, grade, age, gender, smoking status and observational time was collected. Besides, information on blood count analysis was obtained from ambulatory digital charts. On this basis neutrophil-to-lymphocyte ratio (NLR), platelet-to lymphocyte ratio (PLR) and lymphocyte-to-monocyte ratio (LMR) was counted and compared between groups.

**Results:**

NLR, PLR and LMR were shown to be independent prognostic markers of progression in multivariable analysis. The model with stage, grade, age, gender, smoking status and LMR had the highest prognostic values of all models (area under curve [AUC] = 0.756). The cut-off point according to ROC curves for LMR was 3.25. Adding LMR to the baseline model including clinical variables significantly increased area under curve by 0.08 (p = 0.001). NLR and PLR did not increase areas under curve significantly to baseline model.

**Conclusions:**

LMR outperformed NLR and PLR for prediction of progression in patients with non-muscle-invasive bladder cancer receiving BCG immunotherapy. LMR, as an easily obtainable biomarker, should be incorporated to the present risk stratification models.

## Introduction

Bladder cancer (BCa) is the ninth most common cancer worldwide (1). Most of the patients are initially diagnosed with non-muscle invasive BCa (NMIBC), but up to 45% will progress to muscle-invasive disease within 5 years (2, 3). The goal of NMIBC bladder-sparing management is to achieve local control of the disease, and defer or avoid radical cystectomy. At present, the most common adjuvant treatment in intermediate and high-risk patients includes BCG (Bacillus Calmette-Guerin) immunotherapy. Present evidence suggest its efficacy with a 32% decrease in the recurrence rate and 27% in progression rate to muscle-invasive disease (4). It comprises of induction and maintenance phases, lasting up to three years. According to recent evidence, the efficacy of such therapy is based on local and systemic immunological responses (5, 6).

Despite advances in NMIBC management there is a lack of reliable biomarkers that could guide follow-up after TURBT and BCG instillations. Blood-based inflammatory biomarkers such as neutrophil-to-lymphocyte ratio (NLR), platelet-to-lymphocyte ratio (PLR) were introduced as a valuable prognostic markers for urologic cancers, including bladder cancer (7–14), Recent evidence indicates that a lower peripheral lymphocyte-to-monocyte ratio (LMR) is closely associated with worse prognosis in patients with various cancers and could be considered as another easily available and reliable prognostic biomarker (9, 15, 16).

Improvement in risk stratification of NMIBC is particularly important, as could lead to personalized surveillance tailoring, and delineation of disease trajectory, which would result in patient’s quality of life improvement, cost-effectiveness and enhanced oncological outcomes. Therefore, our primary goal was to evaluate and compare the prognostic role of NLR, PLR and LMR in a cohort of patients who underwent BCG-immunotherapy for NMIBC.

## Material and Methods

We retrospectively analyzed the data from our ambulatory and department digital charts of patients with NMIBC who underwent TURBT followed by BCG immunotherapy. Inclusion criteria were as follows: initial BCG therapy starting not earlier than 6 years ago, complete data regarding all pathology reports, complete follow-up cystoscopy and subsequent treatment after recurrence and progression during BCG therapy. We also obtained the information if a patient eventually died due to bladder cancer. We considered patients with medium-risk recurrent low-grade non-invasive (pTa) disease as patients without progression. We excluded patients with sole carcinoma in-situ (only 12 patients) and those intolerant for BCG therapy. The group of patients with both pT1 and Carcinoma in-situ (CIS) (the highest risk group) was combined with pT1 group as only seven patients in this group received BCG therapy (most of these patients underwent early cystectomy). Progression was defined as the disease changing its pathology from non-muscle invasive to muscle-invasive or the recurrence of high-grade disease. Some 173 patients met these criteria, and 61 of these progressed. With propensity score matching we paired each patient with progression to another patient without progression according to age and gender. For three pairs we found additional patients without progression with almost the same propensity score and they were also included in the analysis.

Finally, the database of 125 patients (61 with progression and 64 without progression) was analyzed. All patients received Onco-BCG according to Morales scheme. Follow-up consisted of cystoscopy, cytology, and contrast enhanced computer tomography in time intervals according to EAU guidelines.

### Statistical Analysis

Demographic variables were compared with t-test for continuous variables and Chi-square test for categorical variables. In the case of skewed distributions of data logarithmic transformation was applied to correct for normality and stabilize the variance. We constructed four models based on logistic regression. The first model (with only clinical data) counted the probabilities of each patient for developing progression based on stage, grade, age, gender and smoking status. Subsequent three models comprised the clinical data and one hematological variable. The second model consisted of clinical data and LMR, third model—clinical data and PLR, fourth model clinical data and NLR. Finally, the models nos. 2, 3 and 4 were compared with the baseline model (no. 1) in ROC curves. We considered patients with longer observational times and those who eventually died of bladder cancer more important to our analysis. For this analysis we applied weights to each patient equal to observational times (which started on the day of first BCG dose and ending: on the December of 2020 for those without progression; on the day of progression for those with progression). The highest weight was given to patients who eventually died due to bladder cancer. In addition we analyzed the impact of clinical and hematological variables on progression free survival with Cox regression. Similarly to four logistic regression models we constructed the same four models but analyzed them with Cox regression. Harrell’s C-index for each model was counted. All data were analyzed with Statistica Statsoft package ver 13. P values of less than 5% were considered statistically significant.

## Results

Demographic and clinical characteristics are shown in **Table 1**. As patients were matched according to propensity score counted based on age and gender these two are not different between groups. **Table 2** shows the outcomes of logistic regression with clinical data and LMR (Model no. 2). Odd ratios (OR) show how unfavorable factors worsen the chance of developing progression. For example, patients with pT1 have over four times higher odds for developing progression than patients with pTa. On the other hand, continuous data show the increase in odds when changing its value of one unit. If the value of OR is lower than 1 (like LMR) the odds decrease. Therefore, the increase in LMR of 1 decreases the odds of developing progression of 46% ((1 − 0.54) ∗ 100%).

**Table 1:** Demographic and clinical data of patients in analysis.

		Patients without progression (n = 64)	Patients who progressed (n = 61)	*p* value
Age (years, SD)		69.3 (8.29)	69.6 (6.82)	0.85
Gender (n, %)	Female	10 (55.5)	8 (44.4)	0.68
	Male	54 (50.47)	53 (49.53)	
Smoking status (n, %)	Yes	24 (41.38)	34 (58.62)	0.04
	No	40 (59.7)	27 (40.3)	
Stage (n %)	Ta	36 (67.9)	17 (32)	0.001
	T1	28 (38.8)	44 (61.1)	
Grade (n, %)	LG	42 (60.8)	27 (39.1)	0.01
	HG	22 (39.2)	34 (60.7)	
Observational time (months, SD)		24.6 (19.1)	22.4 (24.2)	0.55
LMR (n, SD)		3.87 (2.1)	2.71 (1.03)	<0.001
PLR (n, SD)		137.7 (77)	133.7 (56)	0.74
NLR (n, SD)		3.07 (2.18)	3.23 (1.88)	0.65

LMR, lymphocyte to monocyte ratio; PLR, platelet to lymphocyte ratio; NLR, neutrophil to lymphocyte ratio.

**Table 2:** Multivariate analysis of second model.

	Variable	OR	CI (+95%)	CI (−95%)	*p* value
Age		0.997	0.985	1.007	0.538
LMR		0.546	0.506	0.590	0.000
Stage	pT1	4.139	3.350	5.113	0.000
Grade	HG	3.205	2.609	3.937	0.000
Gender	Male	1.059	0.802	1.397	0.683
Smoking status	Yes	2.177	1.822	2.601	0.000

Odds ratios refer to: pT1 vs pTa; HG vs LG; male vs female; smokers vs non-smokers. CI, confidence interval; OR, Odds ratio; LMR, lymphocyte-to-monocyte ratio.

**Figure 1:**
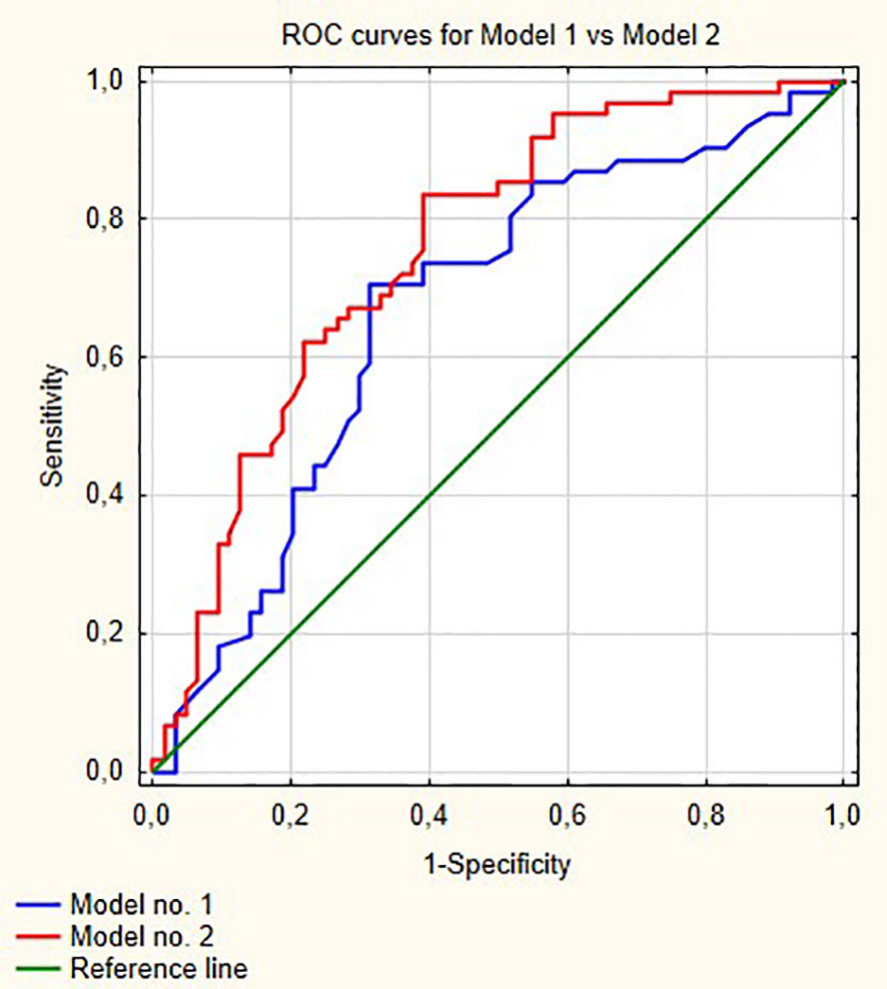
Comparisons of ROC curves for models 1 and 2. AUC for model 1—0.670; model 2—0.756, p = 0.001.

**Figure 2:**
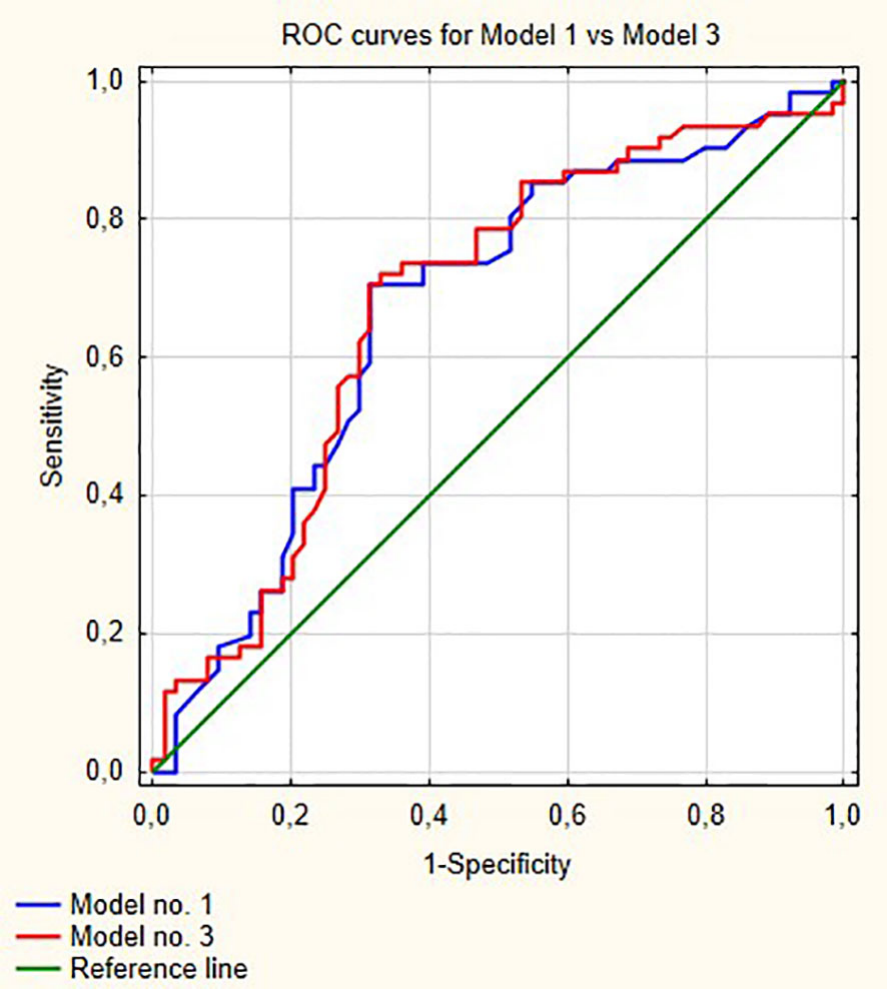
Comparisons of ROC curves for models 1 and 3. AUC for model 1—0.67; model 2—0.677, p = 0.53.

**Figure 3:**
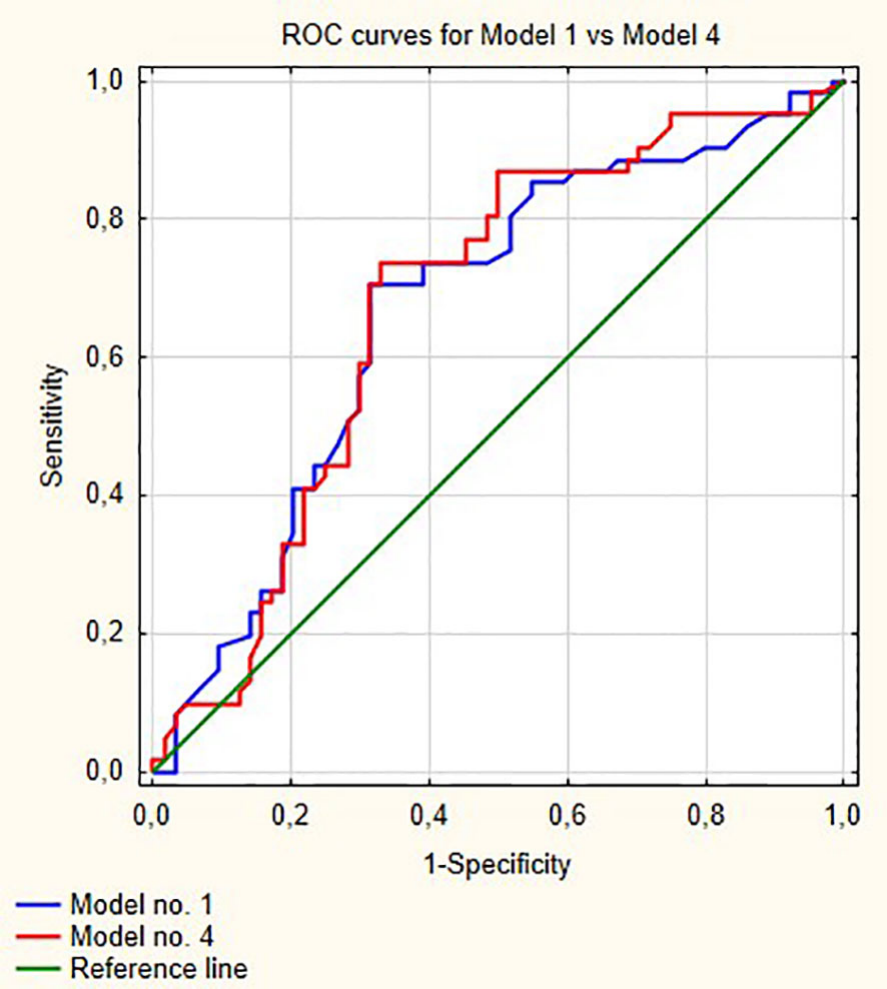
Comparison of ROC curves for models 1 and 4. AUC for model 1—0.670; model 2—0.676, p = 0.58.

**Figure 1** shows ROC curves for models 1 and 2 increase in area under curve (AUC) after adding LMR to the model no. 1 is about 0.08 and this value is statistically significant (p = 0.001). Model numbers 3 and 4 show that NLR and PLR respectively did not increase significantly prognostic values of baseline models (**Figures 2** and **3**).

**Figure 4:**
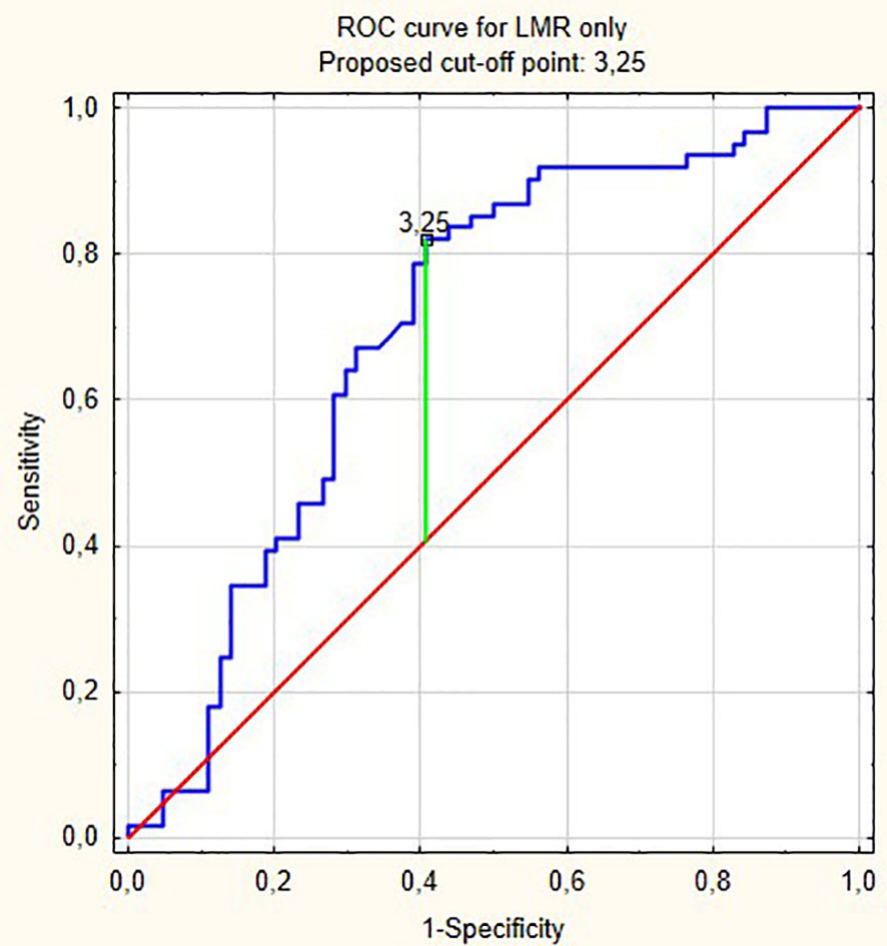
ROC curve for LMR only. Proposed cut-off point: 3,25.

From all hematological biomarkers, LMR showed the highest prognostic value in the prediction of bladder cancer progression in patients receiving BCG immunotherapy. Based on the Youden index, the optimal cut off point of LMR was 3.25, with reverse association with progression (**Figure 4**). **Table 3** shows our group of patients stratified by that cut-off point.

**Table 3:** Clinical parameters in patients divided by LMR cut-off point.

		LMR ≥3.25	LMR <3.25	*p* value
Stage	Ta (n, %)	24 (48)	29 (38.6)	0.3
	T1 (n, %)	26 (52)	46 (61.3)	
Grade	LG	26 (52)	43 (57.3)	0.55
	HG	24 (48)	32 (42.6)	
Gender	Female	9 (18)	9 (12)	0.34
	Male	41 (82)	66 (88)	
Smoking status	No	30 (60)	37 (49)	0.24
	Yes	20 (40)	38 (50.6)	

Stage (HR = 2.26; p = 0.04), grade (HR = 1.42; p <0.01) and smoking status (HR = 1.13; p <0.01) had significant impact on progression free survival in model 1. In models 2, 3 and 4 all clinical variables remained significant. LMR in model 2, PLR in model 3 and NLR in model 4 were also significant with HR = 0.72 (p <0.01), 1.006 (p <0.01) and 1.13 (p <0.01), respectively. Harrell’s C-indices for models 1–4 were: 0.731; 0.783; 0.738 and 0.741 respectively.

## Discussion

In 2009, Spanish Urological Club for Oncological Treatment (CUETO) developed a risk stratification model predicting progression which was based on clinical data for patients with non-muscle invasive bladder cancer receiving BCG immunotherapy (17). However, despite broad implementation, it still lacks of sufficient accuracy. Our study not only focuses on the prognostic value of the included hematological biomarkers but also assesses the added value of each one. It is quite clear that all markers improve, to some extent, the prognostic strength of contemporary models (e.g., CUETO) but our study indicates that among all markers LMR has the highest added value.

In recent years it was shown that not only clinical parameters like stage, grade, presence of carcinoma in situ, age, smoking status and gender are predictors of bladder cancer progression but others also play an important role. Among them are hematological biomarkers like LMR, NLR, PLR, derived neutrophil-to-lymphocyte ratio (dLNR) or even comorbidities, such as diabetes (18, 19). The most studied biomarkers is NLR which showed the prognostic value for invasiveness and progression of bladder cancer, albeit with varying accuracy (20–26). Albayrak et al. emphasized the fact that after adjusting for age NLR lost its prognostic value (27). NLR also predicted progression and recurrence in patients with bladder cancer receiving BCG immunotherapy (13, 28). Yuk et al. showed that in patients receiving BCG immunotherapy NLR was a good predictor of overall survival but not progression-free survival (29).

PLR on the other hand did not show the prognostic value for invasiveness of bladder cancer (20) but the recent meta-analysis underlined its prognostic value for overall survival (30). In patients receiving BCG immunotherapy PLR was not shown to impact progression-free survival (29). Akan et al. showed that PLR predicted progression to muscle invasive disease in patients receiving BCG therapy (31).

LMR was also found as a good prognostic marker for survival in a recent meta-analysis in BCa patients undergoing radical cystectomy (9). Some authors indicate its superiority over NLR in prognostic strength of unfavorable course of disease (16). However, the prognostic value of LMR has not yet been studied in NMIBC patients receiving BCG immunotherapy.

In our multivariable analysis after adjusting for age, stage, grade, gender and smoking status LMR (OR = 0.54; p <0.001), PLR (OR = 1.001; p = 0.04) and NLR (OR = 1.05; p = 0.02) were independent prognostic factors for progression in model nos. 2, 3 and 4, respectively. However, only LMR increased significantly the prognostic value of the model (**Figure 1**) indicating that this variable explains part of the variance which is not elucidated by clinical variables in the baseline model. PLR and NLR also explained such variance but the difference was not significant (**Figures 2** and **3**). According to information in **Table 3** LMR does not correlate with preoperative clinical data and thus explains propensity of developing progression independently.

Despite its pioneer nature, our study has several limitations. First, this is a single-center, retrospective study including only Caucasian patients. Second, we did not adjust the model for the effect of possible other comorbidities or drugs, which could influence biomarkers values. Despite these, we present the first study which determined LMR as independent and the most prognostic factor for progression in NMIBC treated with BCG-immunotherapy.

## Conclusion

LMR is an independent predictor of progression in patients with NMIBC receiving BCG immunotherapy. Its prognostic strength surpasses NLR and PLR in the prediction of progression in patients with bladder cancer receiving BCG immunotherapy.

## Data Availability Statement

The raw data supporting the conclusions of this article will be made available by the authors, without undue reservation.

## Ethics Statement

Ethical review and approval was not required for the study on human participants in accordance with the local legislation and institutional requirements. The patients/participants provided their written informed consent to participate in this study.

## Author Contributions

MA and PR designed the study. MA and PB wrote the paper with input from all authors. PB derived the models and analyzed the data. MA, MK, and BB gathered the data for statistical purposes. AP offered mentorship on every part of the research. All authors contributed to the article and approved the submitted version.

## Funding

This publication was funded by Medical University of Silesia, Katowice, Poland.

## Conflict of Interest

The authors declare that the research was conducted in the absence of any commercial or financial relationships that could be construed as a potential conflict of interest.
